# Bacterial community dynamics during distilled spirit fermentation: influence of mash recipes and fermentation processes

**DOI:** 10.1128/spectrum.01624-23

**Published:** 2023-11-15

**Authors:** Shuang Liu, Isaac V. Greenhut, E. Patrick Heist, Melanie R. Heist, Luke A. Moe

**Affiliations:** 1 Department of Plant and Soil Sciences, University of Kentucky, Lexington, Kentucky, USA; 2 Ferm Solutions Inc, Danville, Kentucky, USA; Wayne State University, Detroit, Michigan, USA

**Keywords:** fermentation, distilled spirits, microbial ecology, lactic acid bacteria

## Abstract

The popularity and production of whiskey have grown dramatically in recent years. During whiskey fermentation, lactic acid bacteria (LAB) are a major concern since they can outcompete yeast and spoil the fermentation. However, some bacteria present in the fermentation could potentially counter this effect and promote fermentation efficiency. To better understand the possible roles bacteria play in yeast-based whiskey fermentations, we examined bacterial community dynamics across fermentation stages and investigated how variation in the mash recipe affects bacterial community composition and fermentation efficiency. To this end, we collected 193 samples from three distilleries at the beginning (Cook/set), middle (Fermentation), and end (Drop) of whiskey fermentation, with six mash recipes sampled from one distillery. We used high-performance liquid chromatography (HPLC) to quantify the contents of sugars, organic acids, and ethanol, which revealed distinct differences between distilleries and mash recipes. High-throughput Illumina Miseq sequencing of the 16S rRNA gene V4 region revealed that bacterial communities shifted toward Firmicutes during the fermentative conversion of sugar to ethanol, especially Lactobacillales. Mash recipes also influenced sugar composition, fermentation efficiency, and microbial dynamics. Recipe-specific operational taxonomic unit (OTU) biomarkers in Drop samples included *Leuconostoc* for corn, *Lactococcus* for wheat, and *Lactobacillaceae_unclassified* for rye, while *Escherichia/Shigella* was associated with sorghum, suggesting potential suppression of LAB.

**IMPORTANCE:**

Production of ethanol from sugars and yeast is an ancient, ostensibly simple process. The source of sugars varies depending on the desired product and can include fruits, vegetables, molasses, honey, or grains, among other things. The source of yeast can be natural in the case of spontaneous ferments, but dry yeast addition is typical for large-scale fermentations. While the polymicrobial nature of some alcoholic fermentations is appreciated (e.g., for wine), most grain-based ethanol producers view microbes, apart from the added yeast, as “contaminants” meant to be controlled in order to maximize efficiency of ethanol production per unit of sugar. Nonetheless, despite rigorous cleaning-in-place measures and cooking the mash, bacteria are routinely cultured from these fermentations. We now know that bacteria can contribute to fermentation efficiency on an industrial scale, yet nothing is known about the makeup and stability of microbial communities in distilled spirit fermentations. The work here establishes the roles of mash recipes and distillery practices in microbial community assembly and dynamics over the course of fermentation. This represents an important first step in appreciating the myriad roles of bacteria in the production of distilled spirits.

## INTRODUCTION

The last two decades have seen an explosion in both the popularity and production of whiskey, and bourbon whiskey in particular, which has seen a 360% increase in production since 2000 (1). Whiskey is produced by aging barreled distillate from yeast-based fermentations, which use a combination of grains as the substrate. Different whiskeys are defined by the proportions of specific grains used for fermentation. Bourbon whiskey, for example, is legally required to contain at least 51% corn in the mash recipe (2), while malted barley predominates in Scotch and Irish whiskeys. Almost all whiskeys contain at least a small amount of malted barley or pure malt, which contributes starch-hydrolyzing enzymes that ensure the availability of fermentable sugars and impart a unique aroma (3). The use of different grain mixtures in the mash recipes can contribute to a variety of flavor and aromatic properties that influence the final product. While corn, wheat, rye, and barley are the most commonly used components of whiskey mash recipes, other grains have been explored for whiskey production, including triticale, rice, and sorghum, the latter of which is typically added as a concentrated sweet molasses produced from crushed sorghum stalks—although distilled spirits produced from non-grain substrates are generally not considered “whiskey” (4).

During whiskey production, the mash (milled grains plus water) is prepared from the designated proportion of grains, which undergo a cooking process prior to being sent to the fermentor. The water used in fermentation can be from municipal or well sources and can include a portion of backset/stillage from a previous batch in a process referred to as “sour mash,” as opposed to “sweet mash,” which does not include backset/stillage. The addition of backset/stillage lowers the pH of the fermentation (5) and is thought to limit bacterial growth during fermentation and ensure consistency between batches. The cooked mash is subsequently cooled, either using an external heat exchanger or in-tank cooling coils, prior to the addition of active dry yeast to initiate fermentation.

While the mash recipe and barrel aging process are largely seen as the key elements imparting the flavor profile to whiskey, distillates comprise a unique chemistry specific to conditions in each fermentation, thus suggesting the intriguing possibility that microbes in the fermentation can also contribute to the overall flavor profile of the final product. Whiskey fermentation shares similarities with fuel ethanol production in that cooked grain mash (typically corn for fuel ethanol production) is used with a high yeast titer in fermentation vessels (6). The high yeast titer is presumed to outcompete any “contaminating” microbes in the fermentor for the available sugars, although bacteria and wild yeast, present in the mash or remaining in the vessel and associated piping, can proliferate during the fermentation.

Bacteria from milled grains, insufficiently cleaned equipment, or incorrectly stored backset are known to colonize fermentation systems (7). Within the industry, external heat exchangers are commonly used and are perhaps the most persistent source of bacterial contamination if not properly sanitized (8). Bacteria have been routinely cultured from samples collected throughout yeast-based fermentation (9), and in extreme cases, one or a few bacterial taxa may dominate a fermentation community and outcompete the yeast, resulting in a “stuck” fermentation. In particular, members of the order Lactobacillales, commonly referred to as lactic acid bacteria (LAB), are well-known to thrive under typical yeast-based fermentation conditions and compete for the same nutrients with yeast (7). However, LAB can have beneficial effects on fermentation in addition to these potential negative impacts. For example, *Lactobacillus amylovorus* was found to contribute to enhancing the growth rate of yeast and ethanol yields in sugarcane fermentation through acetaldehyde cross-feeding (10). Furthermore, another report described the effects of various LAB strains in stabilizing a “stuck” fermentation that was dominated by the model inhibitory strain, *Lactobacillus fermentum* 0315–1 (11). In light of results showing that a highly diverse and stable microbiome is essential to the productivity of an industrial bioenergy ecosystem (12), we sought to explore the ecological factors driving the positive or negative effects of bacterial constituents in distilled spirit fermentation communities.

Distillery-specific sanitization practices, differences in mash recipes and grain sources, and the use of backset/stillage can affect bacterial community composition since they can serve as points of bacterial entry into (or removal from) a fermentation. In particular, the bacterial communities associated with specific mash recipes, their dynamic changes during fermentation, and the effects of these shifts on ethanol yield have not yet been characterized in detail or compared between distilleries. To better understand the contributions of mash recipes and distillery-specific practices on bacterial community structure and fermentation success, we characterized samples from six mash recipes from a single distillery and one mash recipe each from two other distilleries. Samples were collected at different stages during the fermentation to monitor changes in bacterial diversity and community composition, with different batches serving as replicates and mash-only samples (i.e., no yeast added during fermentation) to observe the influence of yeast on the bacterial communities. In addition to the bacterial community composition, we also characterized sugar and organic acid contents, as well as ethanol yield.

## RESULTS

### Mash recipes affect final sugar compositions and fermentation efficiency

**Fig 1:**
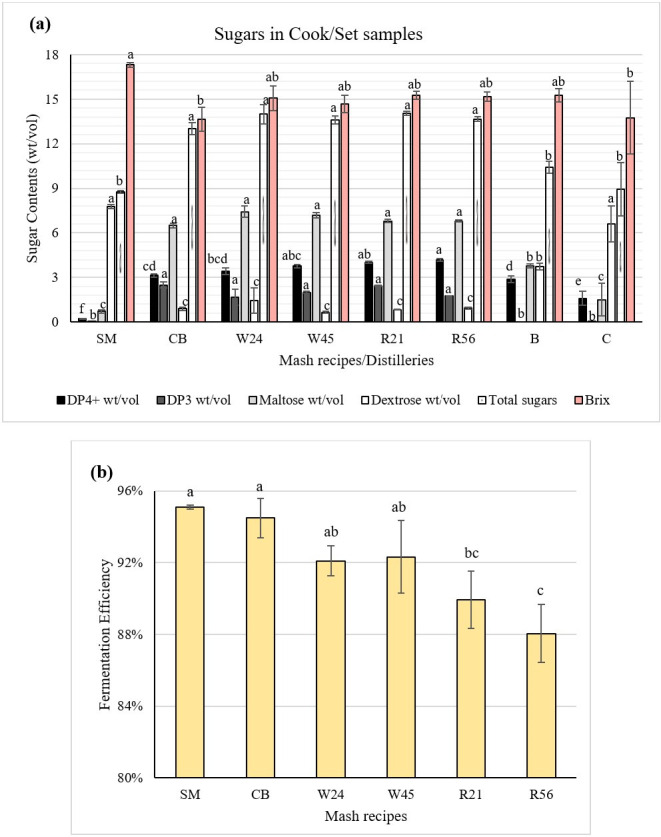
Results of High-performance liquid chromatography (HPLC) analyses. (a) The different sugar contents (DP4+, DP3, maltose, dextrose, and total sugars) and brix of Cook/set samples from distillery A (including SM, CB, W24, W45, R21, and R56 mash recipes), b, and c. (b) The fermentation efficiency of SM, CB, W24, W45, R21, and R56 mash recipes from distillery A. Standard deviation is shown by error bars. The different letters above histograms indicate significant differences in fermentation efficiency between mash recipes.

In order to determine the initial contents and composition of sugars in the cooked mash or sorghum wash [sorghum molasses (SM) were cooked ahead of time and added directly], we collected Cook/set samples at the time of yeast addition. The results showed that Cook/set samples from distilleries B and C contained significantly less total sugar, maltose, DP3, and DP4+ than distillery A samples, but significantly more dextrose, with the exception of distillery A SM (Fig. 1a).

We also compared the brix and sugar compositions from different mash recipes cooked at distillery A, which revealed several striking differences in the sugar contents of the initial substrates. In particular, the SM recipe had significantly higher brix and dextrose than all other mash recipes but lower maltose, DP3, DP4+, and total sugar contents. The corn-barley (CB) mash recipe had the lowest Brix, total sugars, maltose, and DP4+ compared to other mash recipes from distillery A. Wheat mash recipes (W24 and W45) contained the highest relative levels of maltose, while rye mash recipe (R21 and R56) samples contained the highest relative levels of DP4+ (Fig. 1a).

Fermentation efficiency was calculated based on the concentration of ethanol, sugars, lactic acid, and glycerol in the Drop samples. On average, distillery A samples had higher fermentation efficiency (92.0%) than distillery B (84.6%) and C (85.5%). Within distillery A, mash recipes had a significant influence on the fermentation efficiency (*P < 0.05*), with SM (95.1%) and CB (94.5%) showing the highest fermentation efficiency, which was significantly greater than that of mash recipes with rye R21 (89.9%) and R56 (88.0%). Wheat mash recipes had intermediate fermentation efficiency: 92.1% for W24 and 92.3% for W45 (Fig. 1b). Rye mash recipe samples (R21 and R56) had the highest proportion of lactic acid in their Drop samples, comprising 0.35% and 0.31%, respectively. By contrast, the comparatively high-ethanol SM Drop samples only contained 0.02% lactic acid. These data thus implied an inverse relationship between ethanol and lactic acid contents in Drop samples. Taken together, our results indicated that differences in mash recipe composition can influence the contents of component sugars at the beginning of fermentation, as well as the fermentation efficiency, which could be related to microbial dynamics during the fermentation process.

### Predominant operational taxonomic units (OTUs) differ between distilleries

To better understand how differences between mash recipes and distilleries are related to differences in the bacterial constituents and consequently the fermentation efficiency and metabolite accumulation in these yeast-driven fermentation systems, we performed 16S amplicon analysis of samples collected at Cook/set, Fermentation, and Drop by high-throughput sequencing of the V4 hypervariable region of the 16S rRNA gene. Rarefaction curves for samples with OTUs binned at 97% identity showed a plateau at around 1,150 reads per sample, indicating sufficient sequencing depth to characterize the bacterial OTU richness across samples (Fig. S1). Phylum-level classification, with a cut-off of 0.1% relative abundance for distinct groups, revealed four predominant phyla across distillery A samples, including Firmicutes, Proteobacteria, Bacteroidetes, and Actinobacteria. The predominant phyla in distilleries B and C consisted of these same four phyla, as well as a small proportion of Thermotogae (less than 1%) detected in water samples that were not collected from distillery A.

We then determined the 20 most abundant OTUs in each distillery using a cut-off of 1.0% of total abundance for designation as a significant OTU. We found that these 20 predominant OTUs accounted for 81.1%, 78.8%, and 79.3% of all OTUs in distilleries A, B, and C, respectively. Since the most abundant OTU in distillery B (OTU8) and distillery C (OTU2) could not be detected among the top 20 OTUs of other distilleries, we examined the 20 most prevalent OTUs separately for each distillery. Cumulatively, a total of 37 OTUs (Table 1), spanning five phyla, including Firmicutes (18 OTUs), Proteobacteria (11 OTUs), Bacteroidetes (3 OTUs), Actinobacteria (3 OTUs), Thermotogae (1 OTU), and unclassified (1 OTU) comprised all of the top 20 OTUs for all three distilleries combined. Seven of these OTUs (four Lactobacillales in Firmicutes, two Proteobacteria, and one Actinobacteria) could be found among the 20 most abundant OTUs of all distilleries, while all other OTUs among these 37 occurred in samples from only one or two distilleries (Fig. S2). This relatively low overlap in bacterial community composition at the OTU level further emphasized the need for separate analyses for each distillery.

**TABLE 1:** Ranking and taxonomy of the 20 most abundant OTUs from three distilleries

OTU no.	OTU abundance ranking in A (total reads)	OTU abundance ranking in B (total reads)	OTU abundance ranking in C (total reads)	Taxa (phylum; class; order; family; genus)
OTU1	**Top 1 (20,834)^*a*^**	Top 17 (576)	Top 8 (2,674)	*Firmicutes; Bacilli; Lactobacillales; Streptococcaceae; Lactococcus;*
OTU2	–^*b*^	–	**Top 1 (14,813)**	*Bacteroidetes; Flavobacteriia; Flavobacteriales; Flavobacteriaceae; Chryseobacterium;*
OTU3	Top 3 (6,635)	–	–	*Firmicutes; Bacilli; Bacillales; Bacillales_unclassified;*
OTU4	Top 18 (682)	Top 2 (5,500)	Top 3 (5,928)	*Firmicutes; Bacilli; Lactobacillales; Leuconostocaceae; Weissella;*
OTU5	Top 2 (8,900)	Top 9 (1,897)	Top 5 (2,991)	*Proteobacteria; Gammaproteobacteria; Enterobacteriales; Enterobacteriaceae; Enterobacteriaceae_unclassified;*
OTU6	Top 5 (5,872)	–	–	*Firmicutes; Bacilli; Lactobacillales; Lactobacillaceae; Lactobacillaceae_unclassified;*
OTU7	Top 4 (6,083)	Top 5 (3,310)	Top 7 (2,991)	*Proteobacteria; Gammaproteobacteria; Pseudomonadales; Moraxellaceae; Acinetobacter;*
OTU8	–	**Top 1 (9,289)**	–	*Firmicutes; Bacilli; Lactobacillales; Lactobacillaceae; Lactobacillus;*
OTU9	Top 8 (3,139)	Top 11(1,098)	Top 2 (6,420)	*Firmicutes; Bacilli; Lactobacillales; Leuconostocaceae; Leuconostoc;*
OTU10	–	Top 3 (4,358)	Top 4 (3,610)	*Proteobacteria; Betaproteobacteria; Burkholderiales; Burkholderiaceae; Burkholderia;*
OTU11	Top 10 (1,836)	Top 12 (867)	Top 9 (2,558)	*Firmicutes; Bacilli; Lactobacillales; Lactobacillaceae; Lactobacillus;*
OTU12	Top 6 (4,662)	Top 14 (757)	–	*Proteobacteria; Gammaproteobacteria; Enterobacteriales; Enterobacteriaceae; Enterobacteriaceae_unclassified;*
				
OTU13	Top 7 (3,348)	Top 8 (1,914)	–	*Bacteroidetes; Flavobacteriia; Flavobacteriales; Flavobacteriaceae; Chryseobacterium;*
OTU14	–	Top 4 (3,352)	Top 20 (706)	*Firmicutes; Bacilli; Lactobacillales; Lactobacillaceae; Lactobacillus;*
OTU17	Top 9 (3,006)	–	–	*Proteobacteria; Gammaproteobacteria; Enterobacteriales; Enterobacteriaceae; Escherichia/Shigella;*
OTU19		Top 6 (2,577)	Top 14 (1,384)	*Proteobacteria; Betaproteobacteria; Burkholderiales; Comamonadaceae; Comamonadaceae_unclassified;*
OTU20		Top 10 (1,805)	Top 12 (1,689)	*Proteobacteria; Betaproteobacteria; Neisseriales; Neisseriaceae; Uruburuella;*
OTU21	–	Top 7 (2,492)	–	*Firmicutes; Bacilli; Lactobacillales; Lactobacillaceae; Lactobacillus;*
OTU22	Top 11 (1,798)	–	–	*Firmicutes; Bacilli; Lactobacillales; Leuconostocaceae; Leuconostoc;*
OTU23	Top 14 (1,298)	Top 13 (845)	–	*Firmicutes; Bacilli; Lactobacillales; Leuconostocaceae; Weissella;*
OTU24			Top 6 (2,796)	*Firmicutes; Bacilli; Lactobacillales; Lactobacillaceae; Pediococcus;*
OTU25	Top 12 (1,611)			*Firmicutes; Clostridia; Clostridiales; Clostridiaceae_1; Clostridium_sensu_stricto;*
OTU26	Top 13 (1,547)	–	Top 19 (798)	*Firmicutes; Bacilli; Lactobacillales; Streptococcaceae; Lactococcus;*
OTU27	–	–	Top 10 (2,044)	*Actinobacteria; Actinobacteria; Actinomycetales; Corynebacterium; Corynebacterium;*
OTU28	Top 20 (581)	Top 19 (559)	Top 17 (924)	*Actinobacteria; Actinobacteria; Actinomycetales; Micrococcaceae; Arthrobacter;*
OTU30	–	–	Top 11 (1,694)	*Firmicutes; Bacilli; Lactobacillales; Lactobacillaceae; Lactobacillus;*
OTU31	Top 17 (905)	–	Top 18 (872)	*Actinobacteria; Actinobacteria; Actinomycetales; Micrococcaceae; Arthrobacter;*
OTU32	–	–	Top 15 (1,202)	*Firmicutes; Bacilli; Lactobacillales; Enterococcaceae; Enterococcus;*
OTU34	Top 15 (1,143)	–	–	*Proteobacteria; Gammaproteobacteria; Pseudomonadales; Pseudomonadaceae; Pseudomonas;*
OTU35	–	–	Top 13 (1,631)	*Firmicutes; Bacilli; Lactobacillales; Lactobacillaceae; Lactobacillus;*
OTU39	–	–	Top 16 (927)	*Bacteroidetes; Sphingobacteriia; Sphingobacteriales; Sphingobacteriaceae; Sphingobacterium;*
OTU42	Top 19 (648)	–	–	*Proteobacteria; Gammaproteobacteria; Alteromonadales; Pseudoalteromonadaceae; Pseudoalteromonas;*
OTU43	Top 16 (983)	–	–	*Proteobacteria; Gammaproteobacteria; Pseudomonadales; Moraxellaceae; Acinetobacter;*
OTU44	–	Top 20 (548)	–	*Thermotogae; Thermotogae; Petrotogales; Petrotogaceae; Oceanotoga;*
OTU46	–	Top 16 (578)	–	*Firmicutes; Bacilli; Lactobacillales; Enterococcaceae; Enterococcus;*
OTU49	–	Top 18 (563)	–	*Proteobacteria; Betaproteobacteria; Burkholderiales; Burkholderiaceae; Burkholderia;*
OTU72	–	Top 15 (592)	–	*Bacteria_unclassified;*

^*a*^ The rank and total reads # of the top1 OTU in each distillery were bold.

^*b*^ –, not detected. For example, OTU2 was the top1 OTU in distillery C, but it was not detected in distillery A and B.

### Microbiota composition shifts toward Firmicutes during fermentation

In order to characterize changes in microbiota associated with different stages in the fermentation process, we performed non-metric multi-dimensional scaling (NMDS) analysis on samples from different fermentation stages to visualize the shifts in bacterial communities. Since the predominant OTU data (Table 1) revealed considerable differences in bacterial composition between distilleries, we performed NMDS on samples from different distilleries separately.

**Fig 2:**
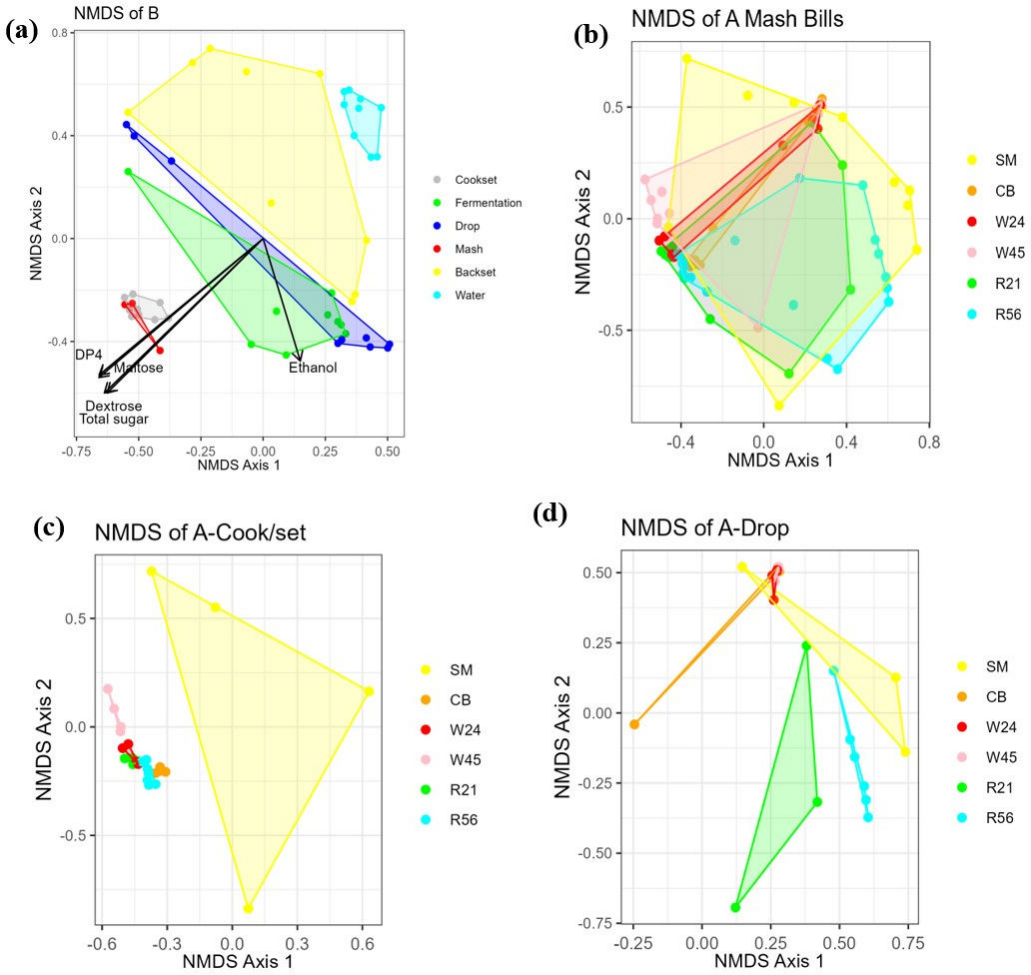
Nonmetric multidimensional scaling plots of bacterial communities. (**a**) Samples from distillery B were grouped by sampling times: Mash, Cook/set, Fermentation, Drop, Backset, and Water. (**b**) Samples from distillery A were grouped by mash recipes (mash bills). (**c**) Cook/set samples from distillery A were grouped by mash recipes. (**d**) Drop samples of distillery A were grouped by mash recipes.

The results of distillery B showed that as sugar decreased, Mash and Cook/set samples clustered together independently from other samples; Fermentation and Drop samples partially overlapped with each other as ethanol accumulated, clustering between early fermentation stages (Mash-Cook/set) and late fermentation stage (Backset). Water samples formed a tight cluster, separate from other samples. Overall, along with the fermentation process, the bacterial communities shifted in the direction of sugar decrease (Fig. 2a). Samples from distillery C showed considerably higher overlap between different fermentation stages, though notably, the bacterial community still showed changes along with fermentation (Fig. S3). In distillery A, regardless of mash recipes and despite the overall co-clustering of Cook/set, Fermentation, and Drop samples, a clear shift can be seen along an axis of decreasing sugars and increasing ethanol and organic acids (Fig. S4). The statistical significance of the NMDS ordinations was analyzed with analysis of molecular variances (AMOVA) and the results are listed in Table S1.

**Fig 3:**
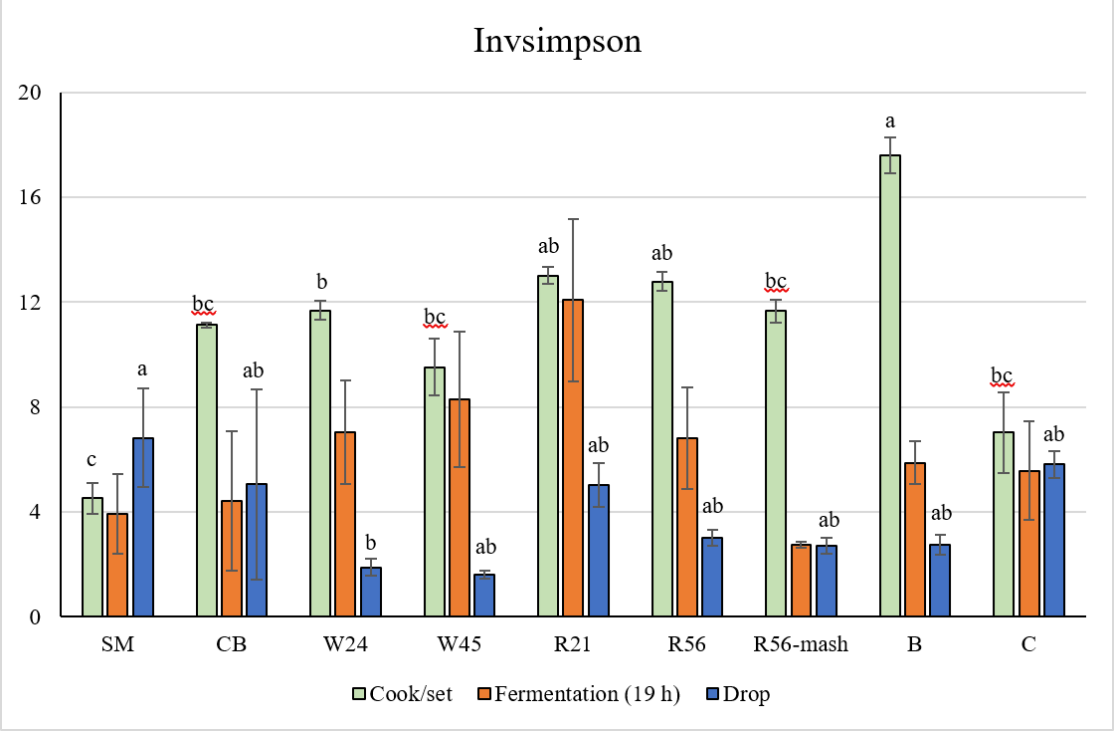
Invsimpson indices of Cook/set, Fermentation, and Drop samples of each mash recipe (SM, CB, W24, W45, R21, and R56) from distillery A and distillery B and C. Error bars are the standard error of the mean. The different letters above the histograms indicate significant differences in inverse Simpson index.

**Fig 4:**
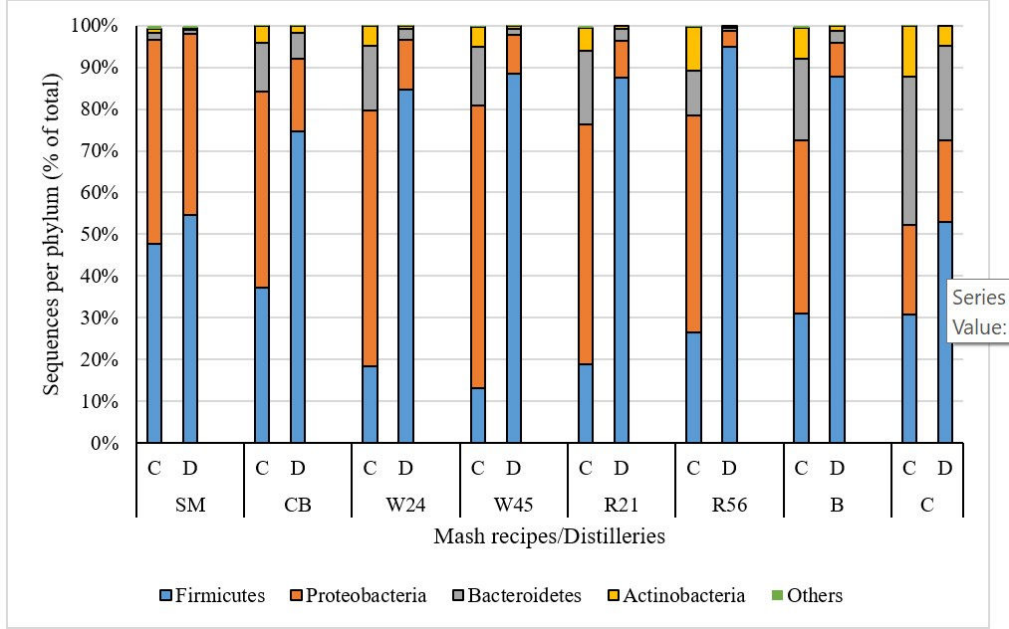
Phylum-level taxonomic distribution of Cook/set (C) and Drop (D) samples from mash recipes SM, CB, W24, W45, R21, R56, and distillery B and C.

**Fig 5:**
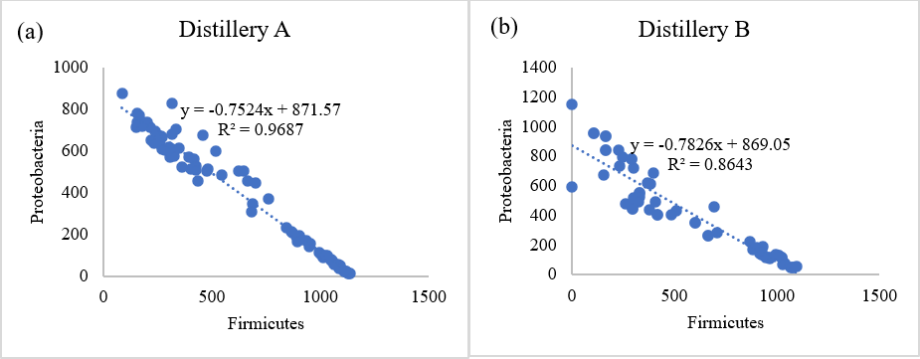
Correlation between the population of Firmicutes and Proteobacteria in mash samples from distillery A (**a**) and distillery B (**b**).

We then used the Inverse Simpson index to estimate diversity, accounting for both the bacterial richness and evenness of their distribution across samples, with higher index values corresponding to greater diversity. The results showed that the bacterial diversity of distillery B tanks exhibited remarkably high diversity in the Cook/set samples, which decreased along with fermentation to its lowest point in Drop samples, similar to that of most distillery A mash recipes (Fig. 3). Next, we examined which phyla participated in this decrease in diversity through the successive stages of fermentation and found a generally steady trend of increasing Firmicutes and decreases in all other phyla over time (Fig. 4). Moreover, correlation analysis showed a strongly negative relationship between Firmicutes and Proteobacteria in distillery A (R^2^ = 0.97, Fig. 5a) and distillery B samples (R^2^ = 0.86, Fig. 5b), with Firmicutes:Proteobacteria ratios in Drop samples of 1.3:1 (SM), 4:3:1 (CB), 7:2:1 (WX), 9.4:1 (WY), 9.7:1 (RX), and 24.9:1 (RY). Interestingly, this correlation was undetectable in distillery C, potentially due to the persistence of significant levels of Bacteroidetes and Actinobacteria in samples from this distillery. Thus, during the conversion of sugars to ethanol, bacterial diversity decreased as Firmicutes proliferated and other taxa, especially Proteobacteria, decreased in abundance. However, among the Drop samples, each mash recipe differed in its Firmicutes: Proteobacteria ratio and fermentation efficiencies (Fig. 1b), which led us to further explore the influence of the mash recipe on bacterial diversity during fermentation.

### Non-grain mash recipe associated with lower bacterial diversity in Cook/set samples

In light of our finding that specific shifts in community composition could be associated with fermentation conditions and sugar conversion, we next investigated how differences in mash recipes could also affect these changes, especially in distillery A, which used six mash recipes: SM (sorghum molasses), CB (corn & barley), W24 and W45 (corn & wheat), R21 and R56 (corn & rye). As in the samples from distilleries B and C, bacterial diversity decreased over fermentation for most distillery A recipes (Fig. 3). However, in the high fermentation efficiency mash recipes (SM and CB), the Inverse Simpson diversity indexes for Drop samples were slightly higher than those of Fermentation samples and even higher than those of Cook/set samples for SM samples, though diversity was remarkably low in Cook/set samples of SM (i.e*.,* non-grain) compared to all other mash recipes.

NMDS analysis of all distillery A samples showed considerable overlap among all mash recipes, suggesting that any effects attributable to mash recipes could be obscured by the effects of the fermentation stage identified in our analyses above (Fig. 2b). To address this issue, we, therefore, examined the clustering patterns of different mash recipes at each fermentation stage separately. Cook/set samples of grain mash recipes generally formed minimally- or non-overlapping, tight clusters, while SM (non-grain) samples were widely separated from all other mash recipe samples (Fig. 2C). Analysis of bacterial composition showed more specific differences between grain and non-grain Cook/set samples: SM had similar proportions of Proteobacteria (48.9%) and Firmicutes (47.8%), whereas other mash recipes had significantly higher percentages of Proteobacteria than Firmicutes at this stage. Grain-based mash recipes also had greater proportions of Bacteroidetes and Actinobacteria, with R21 containing the highest proportion of Bacteroidetes (17.6%) and R56 having the highest proportion of Actinobacteria (10.5%) (Fig. 4).

**Fig 6:**
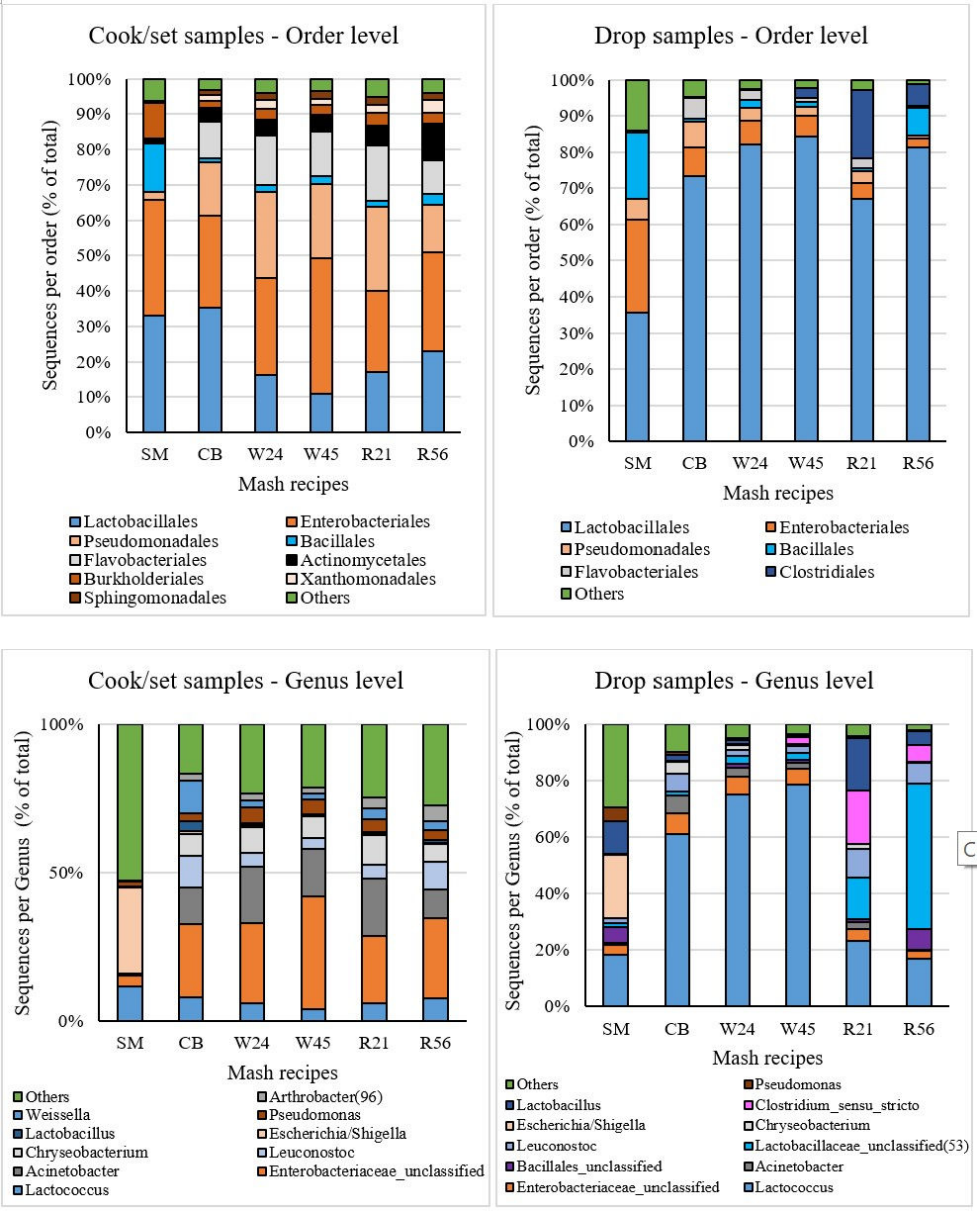
Order and genus-level taxonomic distribution of Cook/set and Drop samples from different mash recipes (SM, CB, W24, W45, R21, and R56) in distillery A.

We further classified OTUs in Cook/set samples with 1.0% or greater abundance at the order- and genus-levels to better understand which specific taxa may significantly contribute to community differences between grain and non-grain mash recipes. At the order level, significant amounts of Pseudomonadales and Xanthomonadales were detected in grain mash recipes but were rare in SM, whereas more Bacillales and Burkholderiales were found in SM than other recipes. SM (33.1%) and CB (35.4%) had the most Lactobacillales, while W24 (16.1%) and W45 (10.8%) had the lowest relative abundance. At the genus level, the most abundant genus in SM samples was *Escherichia/Shigella*, while the most abundant genus among the grain-based mash recipes was *Enterobacteriaceae_unclassified*, although both of these taxa contributed to the preponderance of Proteobacteria. *Acinetobacter*, *Leuconostoc*, and *Chryseobacterium* were present in substantial proportions in grain mash recipes but were barely detectable in SM (Fig. 6). Collectively, these results revealed substantial differences in bacterial community between grain and non-grain mash recipe samples; SM and CB, which showed the highest fermentation efficiency, had the highest abundance of Lactobacillales in the early stage of fermentation.

### LAB taxa in Drop microbiota differ among mash recipes

We examined the clustering patterns of microbiota from each mash recipe in distillery A at the end of the fermentation process in Drop samples using NMDS, which revealed some degree of overlap between SM and all other mash recipes as well as overlap between CB and low-variation (i.e*.,* tightly clustering) wheat samples, whereas samples from different batches of the rye mash recipes formed looser, non-overlapping groups (Fig. 2d).

While Lactobacillales comprised the most prevalent order across Drop samples, each mash recipe had different LAB genera that dominated communities. The proportion of Lactobacillales remained relatively stable in SM but increased dramatically in other mash recipes during the fermentation process, especially in wheat mash recipes (W24 and W45). The highest proportions of *Lactococcus* were detected in the Drop samples of W24 (75.1%), W45 (78.7%), and CB (61.1%), likely accounting for their overlap in NMDS analysis, while this genus was detected in lower percentages in SM (18.3%), R21 (23.2%), and R56 (17.1%). Rye Drop samples (R21 and R56) obviously had more *Lactobacillaceae_unclassified*, *Leuconostoc*, and *Lactobacillus,* in addition to comparatively high levels of the non-LAB Firmicute *Clostridium_sensu_stricto*. Also, notably, *Escherichia/Shigella* persisted in SM samples throughout the fermentation (Fig. 6). These results together suggested that the mash recipe-specific differences, such as the component sugars, could result in enrichment for different Firmicutes genera at the end stages of fermentation, at the expense of Proteobacteria.

Given these clear genus-level differences between mash recipes, we then sought to identify OTU biomarkers in the Cook/set and Drop samples associated with specific mash recipes using Linear discriminant analysis (LDA) effect size (LEfSe) analysis. Based on the mash recipe components, fermentation efficiency, higher order similarity community composition in Cook/set samples (Fig. 6), and high similarity in Drop sample bacterial composition (Fig. 2d and 6), we categorized the six mash recipes into four groups: non-grain (SM), corn (CB), wheat (W24 and W45), and rye (R21 and R56) and identified biomarkers with LDA >3 for Cook/set and Drop samples (Table 2) among the 20 most significant OTUs of each category. Some biomarkers found in the Cook/set samples were also found as biomarkers in the Drop samples, such as OTUs 17 and 42 in the non-grain group, OTU 9 of the corn group, and OTUs 12 and 43 of the wheat group. Among Drop samples, *Leuconostoc* was a biomarker for the corn group; *Lactococcus* was a significant biomarker for the wheat group; and *Lactobacillaceae_unclassified* was a biomarker for the rye group. Notably, no Firmicutes were detected as biomarkers in the Drop samples of SM, though both *Pseudoalteromonas* and *Escherichia/Shigella* were consistent biomarkers at both the beginning and end stages of fermentation. Cumulatively, these findings suggested that the marked differences in bacterial community between grain and non-grain mash recipes persisted from the early stage (Cook/set) to the end (Drop) of fermentation; that is, differences in bacterial community among recipes were also detected in Drop samples (Fig. 6), which could be partially explained by the biomarkers (Table 2) specific to corn, wheat, or rye mash recipes.

**TABLE 2:** Significant biomarkers between different mash recipes with LDA >3 at distillery A

Sample type, mash recipes	No. OTU	Taxonomy (phylum-order-genus)
Cook/set samples		
SM, non-grain	OTU1	*Firmicutes, Lactobacillales*, *Lactococcus*
	OTU3	*Firmicutes, Bacillales, Bacillales_unclassified*
	OTU17	*Proteobacteria, Enterobacteriales*, ***Escherichia/Shigella^a^***
	OTU42	*Proteobacteria, Alteromonadales*, *Pseudoalteromonas*
CB, corn	OTU4	*Firmicutes, Lactobacillales, Weissella*
	OTU5	*Proteobacteria, Enterobacteriales, Enterobacteriaceae_unclassified*
	OTU9	*Firmicutes, Lactobacillales, **Leuconostoc***
	OTU11	*Firmicutes, Lactobacillales, Lactobacillus*
	OTU23	*Same as OTU4*
W24, W45, wheat	OTU7	*Proteobacteria, Pseudomonadales, Acinetobacter*
	OTU12	*Same as OTU5*
	OTU13	*Bacteroidetes, Flavobacteriales, Chryseobacterium*
	OTU34	*Proteobacteria, Pseudomonadales, Pseudomonas*
	OTU43	*Same as OTU7*
R21, R56, rye	OTU26	*Same as OTU1*
	OTU28	*Actinobacteria, Actinomycetales, Arthrobacter*
	OTU31	*Same as OTU28*
Drop samples		
SM, non-grain	OTU17	*Proteobacteria, Enterobacteriales, **Escherichia/Shigella***
	OTU42	*Proteobacteria, Alteromonadales, Pseudoalteromonas*
CB, corn	OTU9	*Firmicutes, Lactobacillales, **Leuconostoc***
	OTU13	*Bacteroidetes, Flavobacteriales, Chryseobacterium*
W24, W45, wheat	OTU1	*Firmicutes, Lactobacillales, **Lactococcus***
	OTU12	*Proteobacteria, Enterobacteriales, Enterobacteriaceae_unclassified*
	OTU43	*Proteobacteria, Pseudomonadales, Acinetobacter*
R21, R56, rye	OTU6	*Firmicutes, Lactobacillales, **Lactobacillaceae_unclassified***
	OTU25	*Firmicutes, Clostridiales, Clostridium_sensu_stricto*

^*a*^ Genus names highlighted in bold were discussed as biomarkers.

### Yeast inhibits the growth of Firmicutes in the early stages of fermentation

**Fig 7:**
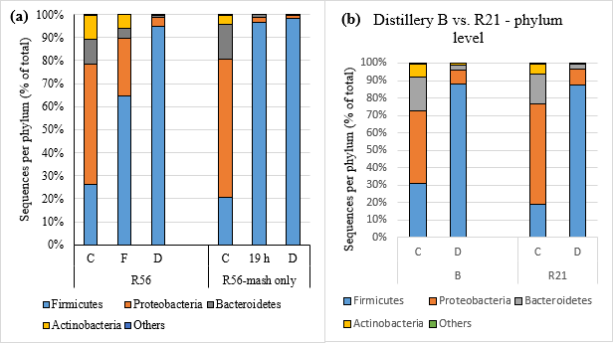
Phylum-level taxonomic distribution. (**a**) Distillery A R56 and R56-Mash-Only samples at Cook/set, fermentation (19 h), and Drop sampling times. (**b**) Distillery B and distillery A R21 samples at Cook/set and Drop sampling times.

Generally, diversity significantly decreased between Fermentation and Drop samples (*P <* 0.05) for all mash recipes in distillery A with the exception of R56-Mash Only, which had no yeast added at the Cook/set stage (Fig. 3). In the Mash Only samples, Inverse Simpson diversity values decreased in Fermentation (19 h) samples to the same level as that in Drop samples, while the proportion of Firmicutes increased to 96.8% at 19 h (Fig. 7a). These findings strongly suggest that yeast likely contributes to mitigating the loss of bacterial diversity and may limit the proliferation of Firmicutes during whiskey fermentation.

## DISCUSSION

Mash recipes of distillery A were grouped into three categories according to high fermentation efficiency (SM and CB), intermediate efficiency (W24 and W45), and low efficiency (R21 and R56); that is, feedstock efficiencies followed the order of: SM and corn > wheat > rye. The comparatively high efficiency of SM could be attributed to the high dextrose content and Brix (fermentable carbohydrate content) in the Cook/set samples (Fig. 1). Sweet sorghum has been proposed as a viable alternative to corn for ethanol production due to its improved lodging resistance in some cultivars that also yield similar or higher levels of ethanol (13). Analysis of alcohol content in beers (the alcoholic liquid produced through fermentation) showed that corn whiskey has a typically higher alcohol content (6.8% alcohol by volume, ABV) than that of rye whiskey (5.8% ABV) (14), and other reports have indicated that the ethanol yields of wheat are significantly higher than those of rye when using the same fermentation method (15).

Distillery B used the same mash recipes as R21 in distillery A, but Cook/set samples from distillery B contained a greater abundance of Firmicutes and fewer Proteobacteria than those from R21 in distillery A (Fig. 7b), and their sugar compositions also differed (Fig. 1a). As noted above, phylum-level composition was similar among Drop samples, both of which had 87.0% Firmicutes, although we observed differences at the order level. Lactobacillales was the largest order in Drop samples of both distillery B (87.2%) and distillery A R21 (67.1%) (data not shown). The second largest order detected in distillery A R21 Drop samples was Clostridiales (19.1%), which was almost undetectable in distillery B Drop samples.

Regardless of distilleries and mash recipes, the bacteria in all samples were predominantly Firmicutes and Proteobacteria, consistent with Li et al.’s (6) study of corn-based fuel ethanol fermentations. Notably, the proportion of Firmicutes increased significantly from the early (Cook/set) stages to the late (Drop) stages while Proteobacteria populations decreased, resulting in a significant inverse correlation between the abundances of these two phyla in distillery A and distillery B (Fig. 5). The increase in Firmicutes was primarily driven by enrichment for the order Lactobacillales (i.e*.*, LAB) since they characteristically ferment carbohydrates into lactic acid. LABs may be the most common bacterial taxa responsible for spoilage during distilled spirit fermentation due to their tolerance for low pH, low oxygen, high temperature, and high ethanol content (16).

Lactic acid bacteria were also detected in Cook/set samples, likely due to carryover from a population of epiphytic or endophytic microbiota of grains (17, 18), and have also been isolated from malt whiskey distilleries (19). In this study, bacterial diversity decreased in all mash recipes between Cook/set and 19 h of Fermentation (Fig. 3), similar to the decline in diversity caused by rapid yeast growth and ethanol accumulation in the first 30–40 h of whiskey fermentation reported in other studies (16, 20). Logarithmic proliferation of LAB starts after 30 to 40 h of fermentation (21), resulting in LAB dominance in Drop samples. In R56 Mash-Only (no yeast) samples, Firmicutes (predominantly LAB) overwhelmed other bacterial taxa within the first 19 h of Fermentation (Fig. 7). By contrast, samples from the same mash recipe with yeast added at Cook/set had a highly diverse (but transient) bacterial community at 19 h between Cook/set and Drop samples. Rapid yeast growth resulted in the lower bacterial diversity of Fermentation samples compared to Cook/set samples, whereas in the absence of added yeast, Firmicutes can generally dominate the bacterial community within a short time. These results thus illustrate the apparent inhibition of Firmicute proliferation (mostly LAB) by yeast in the early stages of fermentation.

Interestingly, the dominant LAB in Drop samples was non-uniform across different mash recipes, with *Lactococcus* comprising the largest proportion in SM, CB, W24, and W45 mash recipes, but *Lactobacillaceae_unclassified* and *Lactobacillus* reaching high abundance in R21 and R56 rye mash recipes. *Lactobacillus* has been described as the predominant LAB in distilleries and fuel ethanol plants (7, 22, 23) but was not consistently detected in this study. *Lactococcus* spp. can also be isolated from fuel ethanol facilities (22) but is more commonly identified in dairy industry facilities (24). In addition, we also detected the LAB genera *Weissella* and *Leuconostoc,* which have been identified in fuel ethanol fermentations (6).

Lactic acid bacteria can be classified as either homofermenters, such as *Lactococcus* and some *Lactobacillus*, which use glycolysis to produce lactic acid as the sole end product, or as heterofermenters, such as *Leuconostoc* and some *Lactobacillus* species, which use the 6-phosphogluconate pathway to produce lactic acid and ethanol or acetic acid (7, 24). Of these two groups, heterofermentative LAB can more severely reduce yeast productivity through competition for sugar substrates than homofermenters (25). In this study, we observed that the homofermentative genus *Lactococcus* was predominant in SM, CB, W24, and W45 Drop samples. In contrast, the R21 and R56 samples contained potentially heterofermentative *Lactobacillaceae_unclassified* and *Lactobacillus* OTUs, in agreement with their comparatively lower fermentation efficiencies and higher lactic acid contents in Drop samples (Fig. S4). Genus-level community structure was similar between W24 and W45 (wheat recipes) and between R21 and R56 (rye recipes) (Fig. 6), which emphasized how mash recipes influence bacterial communities.

Each grain mash recipe had at least one LAB biomarker in Drop samples, while the only two biomarkers in SM Drop samples were Proteobacteria. SM also had the highest fermentation efficiency, significantly higher pH than other mash recipes (Fig. S6), and the fewest LAB in Drop samples. OTU 17 (*Escherichia/Shigella*) was a significant biomarker in both Cook/set and Drop SM samples, suggesting it was introduced in the early stages. Proteobacteria have also been negatively correlated with Firmicutes in fuel ethanol fermentations (6). The low levels of LAB in SM Drop samples here are likely due to the high proportion of *Escherichia/Shigella* (22.4%). By contrast, in the Drop samples of grain mash recipes, *Escherichia/Shigella* accounted for <0.3%, on average. *Escherichia/Shigella* has been reported as a dominant bacterial genus in the fermentation of Maotai-flavored liquor, which includes sorghum in its mash recipe (26). Thus, the sorghum was probably the source of *Escherichia/Shigella,* and its presence likely limited LAB dominance while promoting higher efficiency in fermentation.

Overall, our findings show that the success of Lactobacillales represents the largest characteristic shift in microbiota during whiskey fermentation, although the dominant Lactobacillales subgroup varied among mash recipes. Thus, the mash recipe appears to be the main factor influencing bacterial community composition in distilled spirit fermentation. Moreover, bacterial community composition can vary between fermentations of the same mash recipe in different distilleries, suggesting that distillery-specific practices, such as sanitation, grain source, or other protocols, also contribute to shaping fermentation communities. Findings in this study also support that *Lactococcus* may be less detrimental to yeast growth and ethanol yield than other LABs (*Lactobacillus* and Unclassified *Lactobacillaceae*), while *Escherichia/Shigella* can potentially reduce LAB populations. Since research into the influence of mash recipes on bacterial community dynamics during distilled spirit fermentation remains rare, more studies are needed to understand how the fermentation ecosystem can be engineered for stability to ensure predictability over the course of fermentation and to maximize ethanol efficiency.

## MATERIALS AND METHODS

### Mash sampling

One hundred ninety-three samples were collected from three Kentucky distilleries (hereafter referred to as distillery A, B, or C) using sterile 50 mL conical tubes. Samples comprised a slurry of solid/liquid taken from a spigot on the fermentor at the designated time points (described below) and were from 48 total batches. Samples were frozen and stored at −80°C for later analysis at Ferm Solutions, Inc. (Danville, KY). Of the 48 batches, 28 were from distillery A, 9 were from distillery B, and 11 were from distillery C (Table 3). Distillery A uses a “sweet mash” procedure with in-tank cooling coils, while both distillery B and C use “sour mash” with external heat exchangers.

**TABLE 3:** Explanation of sample names (sample name = mash recipe/distillery + batch no. + sampling time)

Mash recipe/Tank	Batch 1	Batch 2	Batch 3	Batch 4	Batch 5	Batch 6	Mash-Only 1	Mash-Only2
Distillery A								
SM	SM-1-C	SM-2-C	SM-3-C	SM-4-C				
	SM-1-F	NA	SM-3-F	NA				
	SM-1-D4	SM-2-D5	SM-3-D4	SM-4-D5				
CB	CB-1-C	CB-2-C	CB-3-C	CB-4-C				
	CB-1-F	CB-2-F	CB-3-F	CB-4-F				
	CB-1-D5	NA	CB-3-D3	CB-4-D5				
W24	W24-1-C	W24-2-C	W24-3-C	W24-4-C				
	W24-1-F	W24-2-F	W24-3-F	W24-4-F				
	W24-1-D3	W24-2-D3	W24-3-D5	W24-4-D5				
W45	W45-1-C	W45-2-C	W45-3-C	W45-4-C				
	W45-1-F	W45-2-F	W45-3-F	W45-4-F				
	W45-1-D3	W45-2-D5	W45-3-D5	W45-4-D3				
R21	R21-1-C	R21-2-C	R21-3-C	R21-4-C				
	R21-1-F	R21-2-F	R21-3-F	R21-4-F				
	R21-1-D5	R21-2-D3	R21-3-D3	R21-4-D5				
R56	R56-1-C	R56-2-C	R56-3-C	R56-4-C	R56-5-C	R56-6-C	R56-1M-0	R56-2-M0
	R56-1-F	R56-2-F	R56-3-F	R56-4-F	R56-5-F	R56-6-F	R56-1M-19	R56-2M-19
	R56-1-D3	R56-2-D5	R56-3-D5	R56-4-D3	R56-5-D3	R56-6-D5	R56-1M-115	R56-2M-67
Distillery B								
Tank 1	B1-M							
	B1-1-W	B1-2-W	B1-3-W					
	B1-1-B	B1-2-B	B1-3-B					
	B1-1-C	B1-2-C	B1-3-C					
	B1-1-F	B1-2-F	B1-3-F					
	B1-1-D	B1-2-D	B1-3-D					
Tank 2	B2-M							
	B2-1-W	B2-2-W	B2-3-W					
	B2-1-B	B2-2-B	B2-3-B					
	B2-1-C	B2-2-C	B2-3-C					
	B2-1-F	B2-2-F	B2-3-F					
	B2-1-D	B2-2-D	B2-3-D					
Tank 3	B3-M							
	B3-1-W	B3-2-W	B3-3-W					
	B3-1-B	B3-2-B	B3-3-B					
	B3-1-C	B3-2-C	B3-3-C					
	B3-1-F	B3-2-F	B3-3-F					
	B3-1-D	B3-2-D	B3-3-D					
Distillery C								
Tank 1	C1-1-M	C1-2-M	C1-3-M	C1-4-M				
	NA	C1-2-W	C1-3-W	C1-4-W				
	C1-1-B	C1-2 -B	C1-3-B	C1-4-B				
	C1-1-C	C1-2-C	C1-3-C	C1-4-C				
	C1-1-F	C1-2-F	C1-3-F	C1-4-F				
	C1-1-D	C1-2-D	C1-3-D	C1-4-D				
Tank 2	C2-1-M	C2-2-M						
	C2-1-W	C2-2-W						
	C2-1-B	C2-2-B						
	C2-1-C	C2-2-C						
	C2-1-F	C2-2-F						
	C2-1-D	C2-2-D						
Tank −3-	C3-1-M	C3-2-M	C3-3-M					
	NA	C3-2-W	C3-3-W					
	C3-1-B	C3-2-B	C3-3-B					
	C3-1-C	C3-2-C	C3-3-C					
	C3-1-F	C3-2-F	C3-3-F					
	C3-1-D	C3-2-D	C3-3-D					
Tank 4	C4-1M	C4-2M						
	C4-1W	C4-2W						
	C4-1B	C4-2B						
	C4-1C	C4-2C						
	C4-1F	C4-2F						
	C4-1D	C4-2D						

The 28 batches from distillery A included six mash recipes: SM (100% sorghum molasses, referred to as sorghum “wash”), CB (82% corn, 10% smoked barley, and 8% malt), W24 (64% corn, 24% wheat, and 12% malt), W45 (51% corn, 45% wheat, and 4% malt), R21 (75% corn, 21% rye, and 4% malt), and R56 (33% corn, 56% rye, and 11% malt). Samples were collected at three time points for each batch in distillery A: the first time point was at Cook/set (C) at 0 h, when yeast was added to the cooked mash to initiate fermentation; the second time point was at 19 h of Fermentation (F); and the third time point was at Drop (D) when the fermentation was completed. Distillery A had three different potential drop times due to the timing of initiation and facility staffing: D3 represents 67 h (3-day) fermentation, D4 represents 92 h (4-day) fermentation, and D5 represents 115 h (5-day) fermentation. Drop time typically does not impact ethanol yield. To determine how the yeast affects the bacterial community during fermentation, two batches of “mash-only” samples (i.e*.,* no yeast was added) were performed using the R56 mashbill, and each of these batches was sampled three times: at 0 h, 19 h, and 115 h.

The nine batches from distillery B were fermented in three separate tanks (three batches per tank): Tank 1 (B1), Tank 2 (B2), and Tank 3 (B3), and all fermentations from distillery B used the same mash recipe: 75% corn, 21% rye, and 4% malt. The 11 batches from distillery C were fermented in four tanks: Tank 1 (C1; four batches), Tank 2 (C2; two batches), Tank 3 (C3; three batches), and Tank 4 (C4, two batches), all of which used the same mash recipe: 75% corn, 13% rye, and 12% malt. Six samples were collected from each batch in distilleries B and C: Mash (M) samples collected from cooked grains; Water (W) used for cooking; Backset (B) samples; Cook/set (C) samples; 19 h of Fermentation (F) samples; and Drop (D) samples. All samples are listed in Table 3.

### High-performance liquid chromatography (HPLC) analyses

Contents of DP4+ (carbohydrates that contain four or more linked glucose units), DP3 (carbohydrates that contain three linked glucose units), maltose, dextrose, acetic acid, glycerol, lactic acid, and ethanol were analyzed by HPLC using a Shimadzu LT-20AT (Shimadzu USA, Canby, OR). The detailed settings and procedures were the same as previously reported (6). Brix, which is commonly used to measure the dissolved sugar content in a liquid solution, was determined with a brix meter using refractive index. The remaining samples were transported on ice to the University of Kentucky and stored at −80°C for future analysis.

### Calculation of fermentation efficiency

The efficiency of fermentation was calculated using the same method as that of commercial distilleries, using the following equation:


$$FE(\%)=\frac{E\times 1.9553}{DP4\times 1.099+DP3\times 1.071+DP2\times 1.053+DP+Lac+Gly\times 0.9782+E\times 1.9553}\times 100\%$$

Abbreviations: FE (%), fermentation efficiency (%); E, ethanol concentration (wt/vol); DP4, DP4 concentration (wt/vol); DP3, DP3 concentration (wt/vol); DP2, maltose concentration (wt/vol); DP, dextrose concentration (wt/vol); Lac, lactic acid concentration (wt/vol); Gly, glycerol concentration (wt/vol). Fermentation efficiency was determined in Drop samples.

### DNA extraction and Illumina Miseq sequencing

Total bacterial DNA was extracted with a GenElute Bacterial Genomic DNA Kit (Sigma-Aldrich, St. Louis, MO) following the manufacturer’s protocol with minor modifications: to avoid grain clogging the columns, a double volume of lysis reagent (Proteinase K and Lysis Solution C) was used to preprocess samples, and only liquid lysates were transferred to the binding column. The extracted DNA was stored at −20°C prior to barcoding by PCR. The V4 regions of the 16S rRNA genes were amplified with dual-index, paired-end read PCR primers with a high-fidelity polymerase (Accuprime; Invitrogen) developed by Kozich et al. (27). Reagents, reaction conditions, thermal cycler program for PCR amplification, and agarose gel confirmation were the same as described by Li et al. (6). PCR products from distillery A samples were sent to the BioFrontiers Core Facility (University of Colorado, Boulder, CO); DNA extracted from distillery B and C samples was sent to the Microbial Systems Molecular Biology Laboratory (MSMBL; University of Michigan, Ann Arbor, MI). MSMBL used a high-fidelity polymerase (Accuprime; Invitrogen), as in Kozich et al. (27), for PCRs with the same PCR conditions used by our lab (6). Thermal cycling parameters were as follows: an initial 5 min at 95°C; 30 cycles of 20 s at 95°C, 15 s at 55°C, and 5 min at 72°C; and a final extension of 72°C for 10 min. Both facilities used SequalPrep Normalization Plate Kits (Thermo Fisher Scientific) to clean and normalize the amplicons. Pooled and quantified amplicon libraries were sequenced (2 × 250 bp) on the Illumina MiSeq platform, with FASTQ files provided as output. Eight samples with insufficient read counts for subsequent analysis were sequenced again at the University of Kentucky HealthCare Genomics Core Laboratory using the same protocols and normalization kits in the original run.

### Analysis of DNA sequencing results

All sequence data were processed using mothur (http://www.mothur.org) version 1.43.0 following the published standard operating procedure (24 June 2019) (13). A total of 7,867,094 sequence reads were generated from the 193 samples after contig assembly. More than 4 million (4,879,181) bacterial 16S rRNA gene V4 region sequences were generated after SILVA-alignment, de-noising, and removal of chimeric and non-bacterial sequences (i.e*.*, chloroplast, mitochondria, unknown, archaea, and eukaryota). The number of sequences per sample ranged from 1,150 to 252,377, with a median number of 22,246. Samples were randomly sub-sampled to 1,150 sequences for normalization. All samples had over 98.6% Good’s coverage, and sequences were clustered into a total of 1,050 OTUs at a 97% sequence similarity. Mothur analysis produced data metrics or rarefaction curves, the Inverse Simpson index (alpha-diversity), non-metric multidimensional scaling (NMDS) ordinations (beta-diversity), and relative abundance at different bacterial taxonomy levels. Linear discriminant analysis effect size analysis was applied to Cook/set and Drop samples, respectively, in distillery A.

### Data visualization

Microsoft Excel version 2016 (Microsoft Corporation, Redmond, WA) was used to generate scatter charts, bar charts, and line charts to visualize rarefaction curves, HPLC data, the Inverse Simpson index, relative abundance at different bacterial taxonomy levels, and correlations between different bacteria. One-way Analysis of Variance (ANOVA) analyses with R version 3.5.1 were performed on HPLC data, and the Inverse Simpson index NMDS ordination plots were produced with R version 3.5.1.

## Data Availability

Raw sequence data were deposited to the sequence read archive under BioProject ID PRJNA824034.

## SUPPLEMENTAL MATERIAL

Fig. S1 to S5, Table S1 spectrum.01624-23-s0001.pdf Supplemental material.

ASM does not own the copyrights to Supplemental Material that may be linked to, or accessed through, an article. The authors have granted ASM a non-exclusive, world-wide license to publish the Supplemental Material files. Please contact the corresponding author directly for reuse.
