# Supplementary material for: Bacterial community dynamics during distilled spirit fermentation: influence of mash recipes and fermentation processes
Source: Microbiol Spectr. 2023 Nov 15;11(6):e01624-23. doi: 10.1128/spectrum.01624-23 (PMC10714749; doi:10.1128/spectrum.01624-23)
Supplement: Fig. S1 to S5, Table S1 — Supplemental material. [file spectrum.01624-23-s0001.pdf]

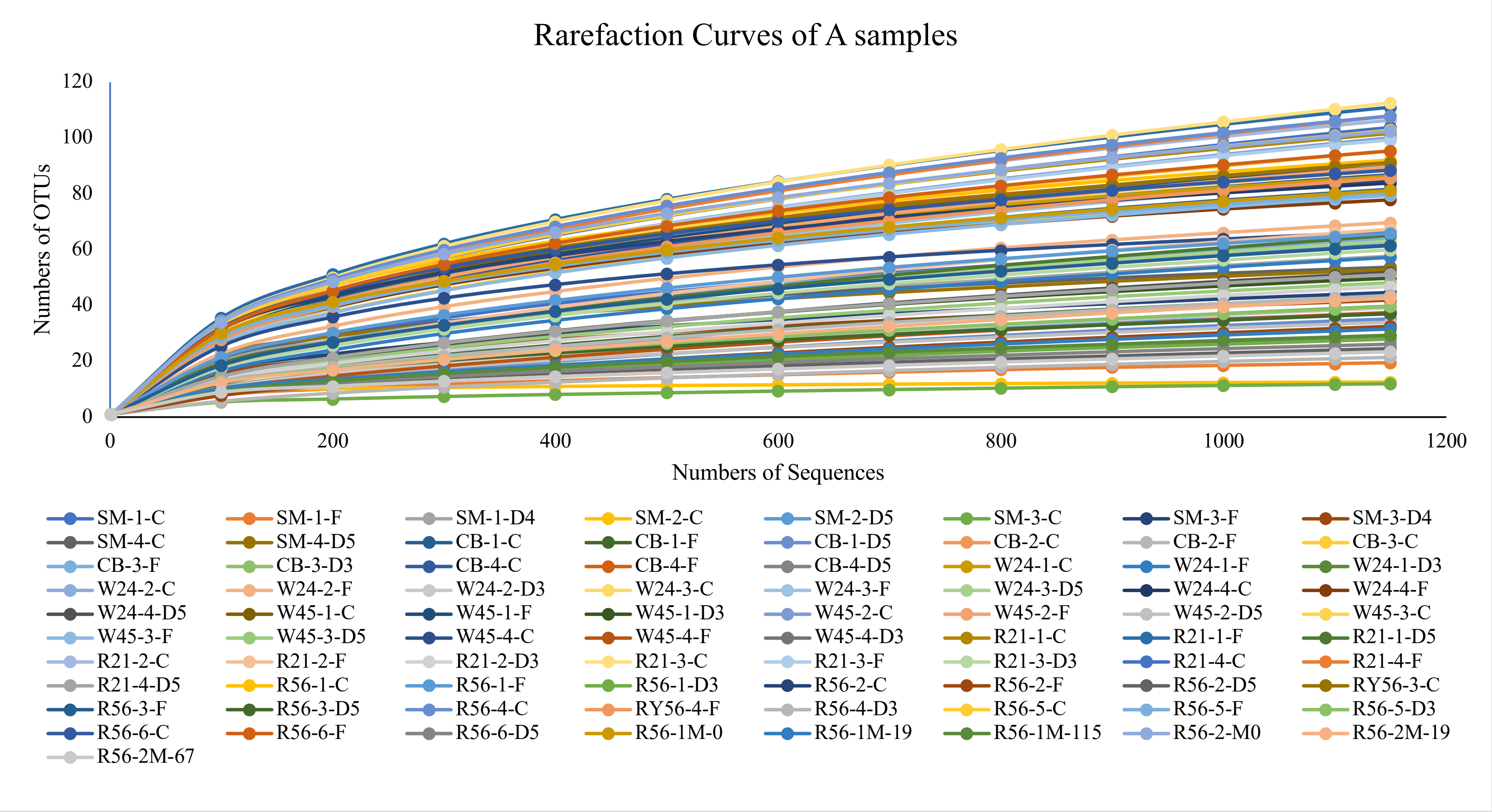
Rarefaction Curves of A samples
Numbers of OTUs
Numbers of Sequences
0
20
40
60
80
100
120
200
400
600
800
1000
1200
SM-1-C
SM-1-F
SM-1-D4
SM-2-C
SM-2-D5
SM-3-C
SM-3-F
SM-3-D4
SM-4-C
SM-4-D5
CB-1-C
CB-1-F
CB-1-D5
CB-2-C
CB-2-F
CB-3-C
CB-3-F
CB-3-D3
CB-4-C
CB-4-F
CB-4-D5
W24-1-C
W24-1-F
W24-1-D3
W24-2-C
W24-2-F
W24-2-D3
W24-3-C
W24-3-F
W24-3-D5
W24-4-C
W24-4-F
W24-4-D5
W45-1-C
W45-1-F
W45-1-D3
W45-2-C
W45-2-F
W45-2-D5
W45-3-C
W45-3-F
W45-3-D5
W45-4-C
W45-4-F
W45-4-D3
R21-1-C
R21-1-F
R21-1-D5
R21-2-C
R21-2-F
R21-2-D3
R21-3-C
R21-3-F
R21-3-D3
R21-4-C
R21-4-F
R21-4-D5
R56-1-C
R56-1-F
R56-1-D3
R56-2-C
R56-2-F
R56-2-D5
RY56-3-C
R56-3-F
R56-3-D5
R56-4-C
RY56-4-F
R56-4-D3
R56-5-C
R56-5-F
R56-5-D3
R56-6-C
R56-6-F
R56-6-D5
R56-1M-0
R56-1M-19
R56-1M-115
R56-2-M0
R56-2M-19
R56-2M-67

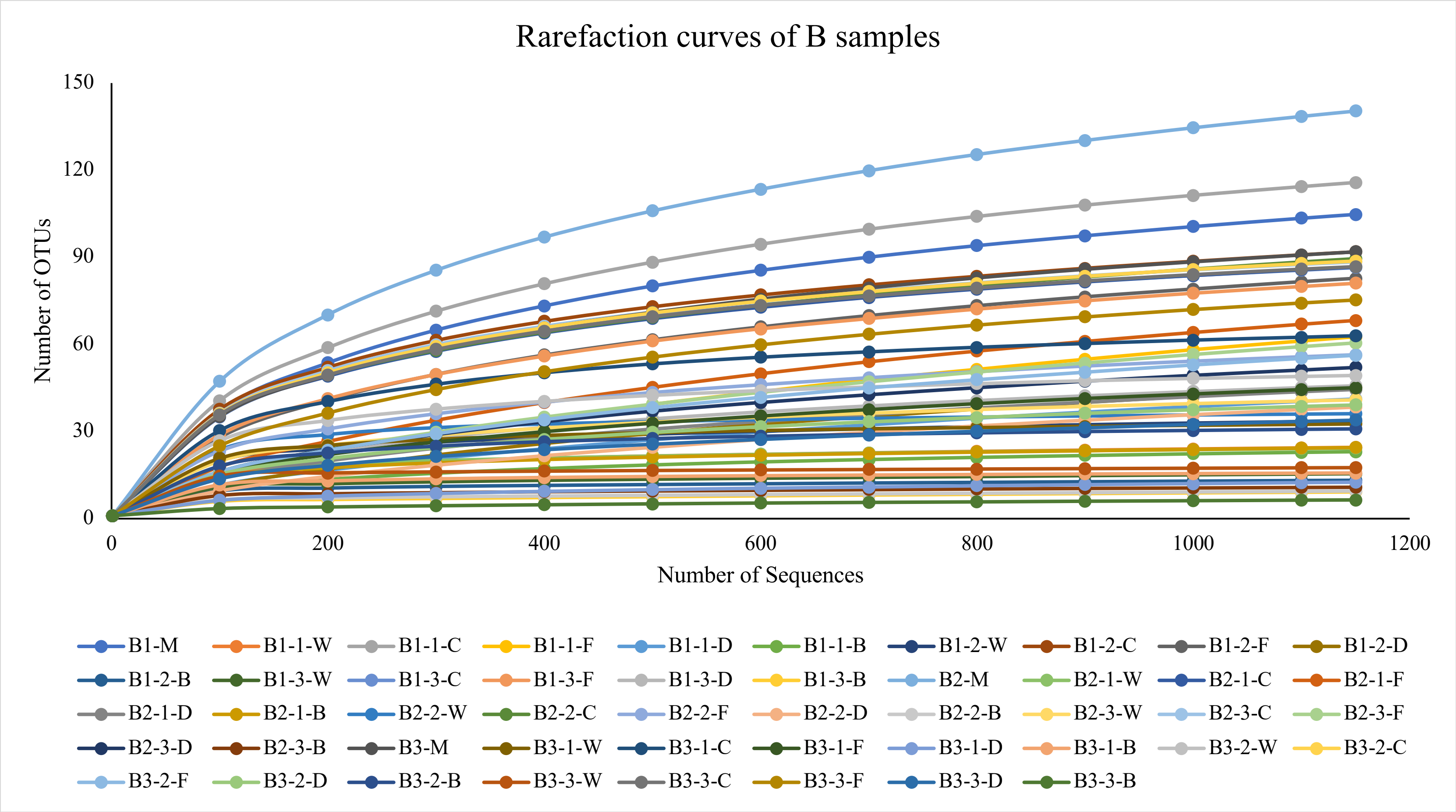
Rarefaction curves of B samples
Number of OTUs
Number of Sequences
0
30
60
90
120
150
0
200
400
600
800
1000
1200
B1-M
B1-1-W
B1-1-C
B1-1-F
B1-1-D
B1-1-B
B1-2-W
B1-2-C
B1-2-F
B1-2-D
B1-2-B
B1-3-W
B1-3-C
B1-3-F
B1-3-D
B1-3-B
B2-M
B2-1-W
B2-1-C
B2-1-F
B2-1-D
B2-1-B
B2-2-W
B2-2-C
B2-2-F
B2-2-D
B2-2-B
B2-3-W
B2-3-C
B2-3-F
B2-3-D
B2-3-B
B3-M
B3-1-W
B3-1-C
B3-1-F
B3-1-D
B3-1-B
B3-2-W
B3-2-C
B3-2-F
B3-2-D
B3-2-B
B3-3-W
B3-3-C
B3-3-F
B3-3-D
B3-3-B

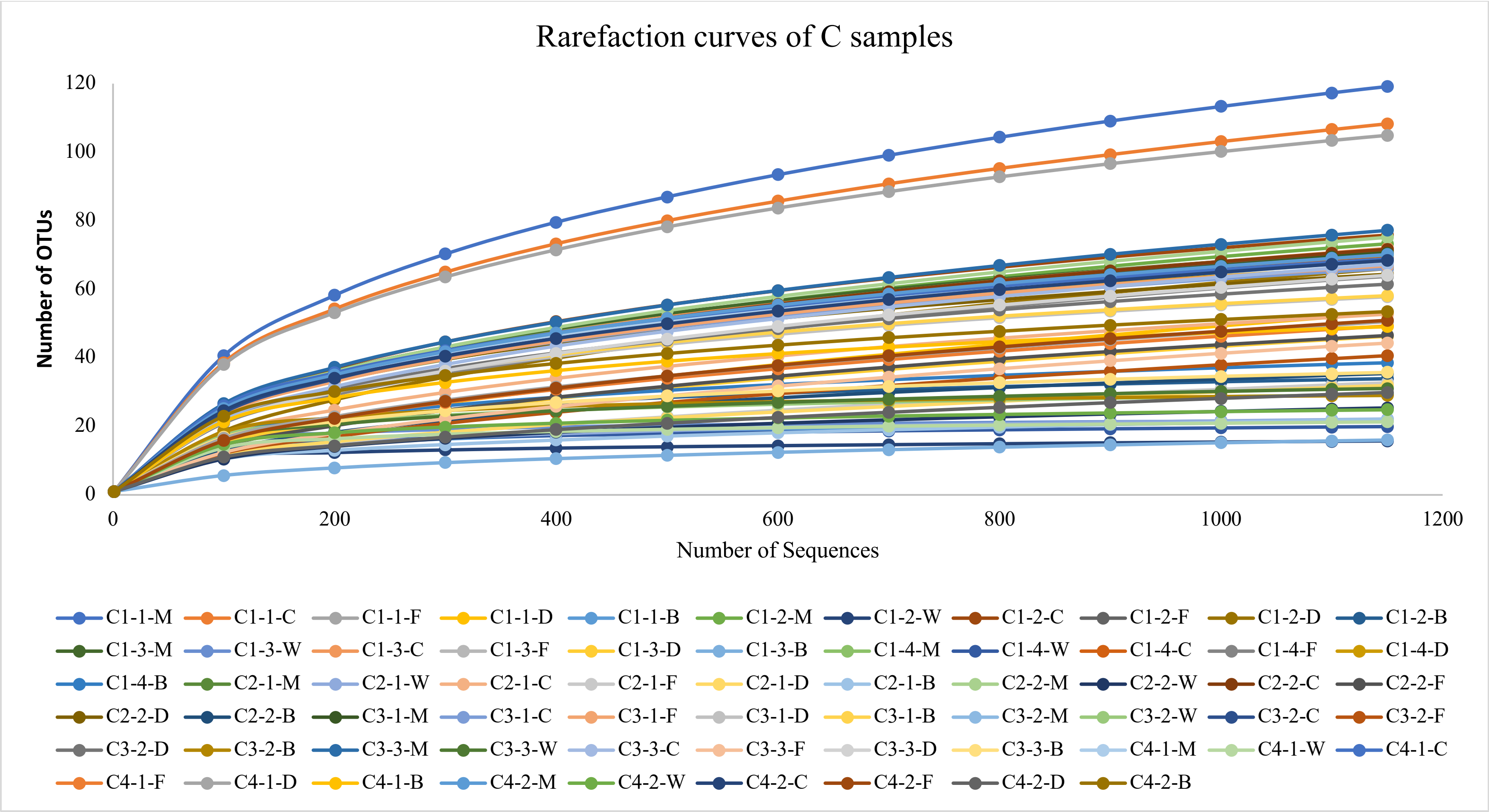

Figure S1. Rarefaction curves based on 16S rRNA gene sequence for mash bills/tanks from distilleries A, B, and C. The OTUs were defined at 97% similarity level.

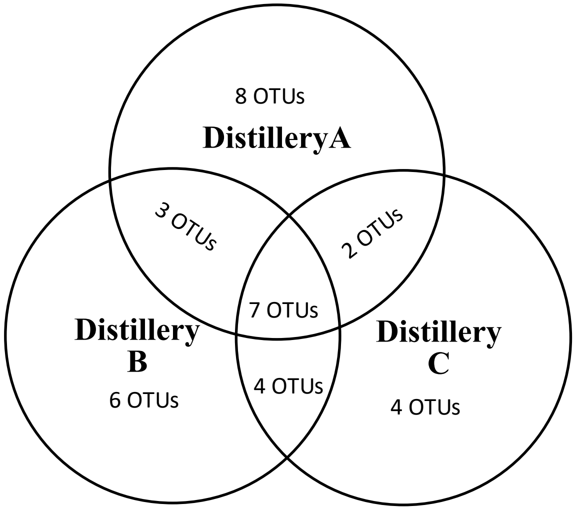

Figure S2. Venn diagram of the number of predominant OTUs identified from Distillery A, B, and C.

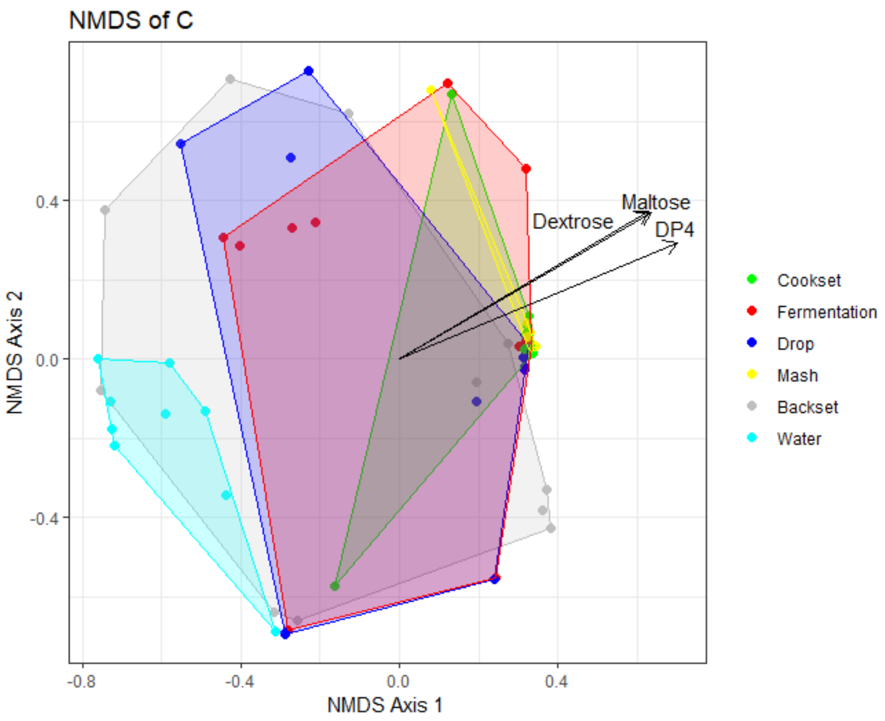

Figure S3. Nonparametric multidimensional scaling plots (NMDS) of bacterial communities from distillery C grouped by different samples: Mash, Cook/Set, Fermentation, Drop, Backset, and Water.

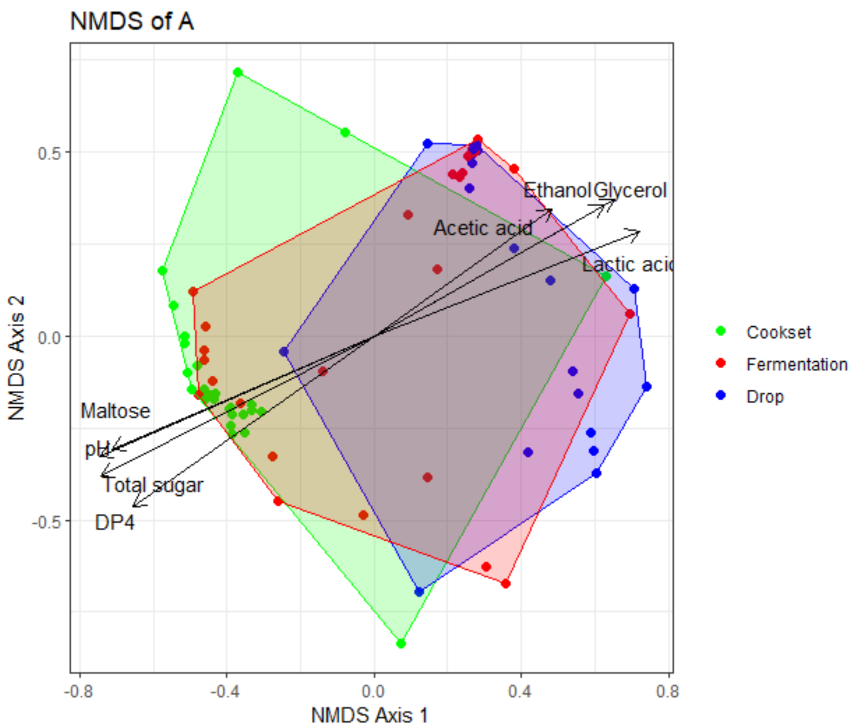

Figure S4. Nonparametric multidimensional scaling plots (NMDS) of bacterial communities from distillery A grouped by sampling times: Cook/Set, Fermentation, and Drop.

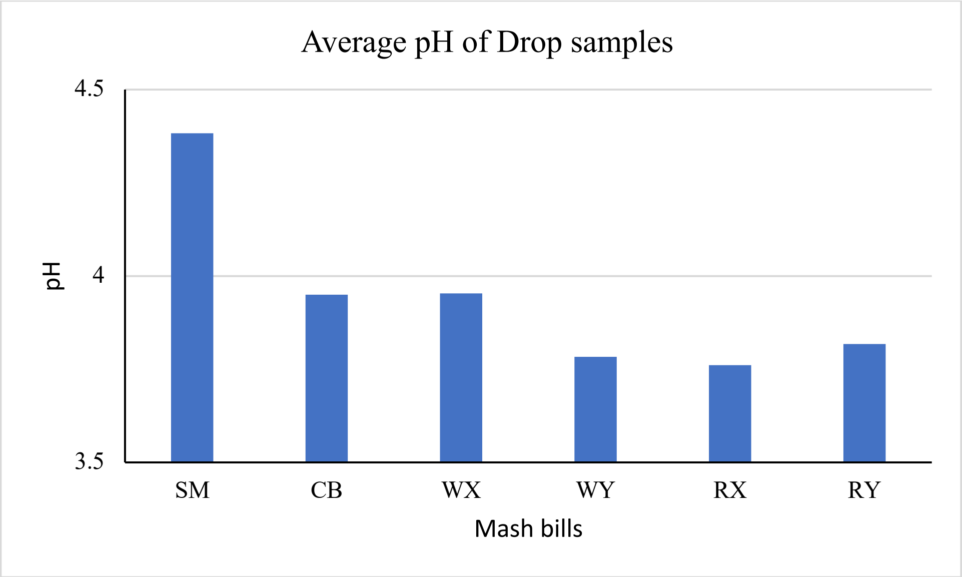

Figure. S5 The average pH of Drop samples of different mash recipes in distillery A.

Table S1 Multiple comparisons of NMDS ordinations.

Distillery A		Distillery B		Distillery C	
Mash Recipes	p-Value	Fermentation Stages	p-Value	Fermentation Stages	p-Value
W24 vs. R21	0.137	Backset vs. Cookset	**<0.001**	Backset vs. Cookset	**0.002**
W24 vs. W45	0.401	Backset vs. Drop	**0.009**	Backset vs. Drop	0.706
W24 vs. CB	0.533	Backset vs. Fermentation	**<0.001**	Backset vs. Fermentation	0.102
W24 vs.SM	**<0.001**	Backset vs. Mash	**<0.001**	Backset vs. Mash	**0.001**
W24 vs. R56	**0.022**	Backset vs. Water	**<0.001**	Backset vs. Water	**0.001**
R21 vs. W45	0.066	Cookset vs. Drop	**<0.001**	Cookset vs. Drop	**0.041**
R21 vs. R56	0.072	Cookset vs. Fermentation	**<0.001**	Cookset vs. Fermentation	**0.011**
R21 vs. CB	0.182	Cookset vs. Water	**<0.001**	Cookset vs. Water	**<0.001**
R21 vs. SM	**<0.001**	Drop vs. Mash	**0.003**	Drop vs. Mash	**0.005**
W45 vs. CB	0.126	Drop vs. Water	**<0.001**	Drop vs. Water	**<0.001**
W45 vs. SM	**<0.001**	Fermentation vs. Mash	**<0.001**	Fermentation vs. Mash	**0.001**
W45 vs. R56	**0.016**	Fermentation vs. Water	**0.001**	Fermentation vs. Water	**<0.001**
SM vs. R56	**<0.001**	Mash vs. Water	**0.004**	Mash vs. Water	**<0.001**
SM vs. CB	**<0.001**	Cookset vs. Mash	0.114	Cookset vs. Mash	0.928
R56 vs. CB	**0.011**	Drop vs. Fermentation	0.056	Drop vs. Fermentation	0.412
